# Recent Advances in Pipeline Monitoring and Oil Leakage Detection Technologies: Principles and Approaches

**DOI:** 10.3390/s19112548

**Published:** 2019-06-04

**Authors:** Mutiu Adesina Adegboye, Wai-Keung Fung, Aditya Karnik

**Affiliations:** 1 Communications and Autonomous Systems Group, Robert Gordon University, Aberdeen AB10 7GJ, UK; m.adegboye@rgu.ac.uk; 2 School of Engineering, Robert Gordon University, Aberdeen AB10 7GJ, UK; a.karnik@rgu.ac.uk

**Keywords:** leakage, leak detection, leak characterisation, leak localisation, pipelines, wireless sensor networks

## Abstract

Pipelines are widely used for the transportation of hydrocarbon fluids over millions of miles all over the world. The structures of the pipelines are designed to withstand several environmental loading conditions to ensure safe and reliable distribution from point of production to the shore or distribution depot. However, leaks in pipeline networks are one of the major causes of innumerable losses in pipeline operators and nature. Incidents of pipeline failure can result in serious ecological disasters, human casualties and financial loss. In order to avoid such menace and maintain safe and reliable pipeline infrastructure, substantial research efforts have been devoted to implementing pipeline leak detection and localisation using different approaches. This paper discusses pipeline leakage detection technologies and summarises the state-of-the-art achievements. Different leakage detection and localisation in pipeline systems are reviewed and their strengths and weaknesses are highlighted. Comparative performance analysis is performed to provide a guide in determining which leak detection method is appropriate for particular operating settings. In addition, research gaps and open issues for development of reliable pipeline leakage detection systems are discussed.

## 1. Introduction

The use of pipeline is considered as a major means of conveying petroleum products such as fossil fuels, gases, chemicals and other essential hydrocarbon fluids that serve as assets to the economy of the nation [1]. It has been shown that oil and gas pipeline networks are the most economical and safest mean of transporting crude oils and they fulfill a high demand for efficiency and reliability [2,3]. For example, the estimated deaths due to accidents per ton-mile of shipped petroleum products are 87%, 4% and 2.7% higher using truck, ship and rail, respectively, compared to using pipelines [4]. However, as transporting hazardous substances using miles-long pipelines has become popular across the globe in recent decades, the chance of the critical accidents due to pipeline failures increases [5]. The causes of the failures are either intentional (like vandalism) or unintentional (like device/material failure and corrosion) damages [6,7], leading to pipeline failure and thus resulting in irreversible damages which include financial losses and extreme environmental pollution, particularly when the leakage is not detected in a timely way [8,9].

**Figure 1:**
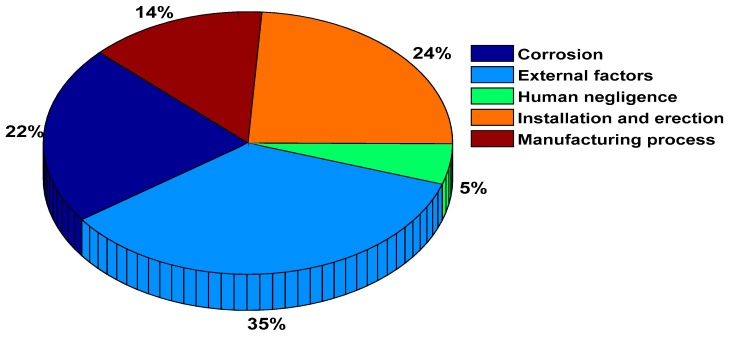
A pie chart of the statistics of the sources of pipeline failure. Data is obtained in [12].

The average economic loss due to incidents of pipeline leakages is enormous [10]. Over the past three decades, pipeline accidents in USA damaged property which costed nearly $7 billion, killed over 500 people and injured thousands [11]. For example, the incident of pipeline explosion in the community of San Bruno, California, USA on September 6, 2010 killed eight people, and injured more than fifty [11]. In a similar incident of pipeline defect that occurred in Michigan, USA on July 26, 2010, more than 840,000 gallons of crude oil spilled into Kalamazoo River with estimated cost of $800 million [11]. The causes of pipeline damage vary. Figure 1 shows a pie chart that illustrates statistics of the major causes of pipelines failure which include pipeline corrosion, human negligence, defects during the process of installation and erection work, and flaws occurring during the manufacturing process and external factors [12].

Based on these statistics, incidents of pipeline leakage are hard to entirely avoid as the sources of failures are diverse. However, in order to reduce the impacts of oil spillage on society it is very important to monitor pipelines for the timely detection of leakage or even leak prediction, as early detection of leaks will allow quick responses to stop oil discharge and proper pipeline maintenance. Hence, it is possible to reduce the loss rate, injuries and other serious societal and environmental consequences due to the pipeline failures.

**Figure 2:**
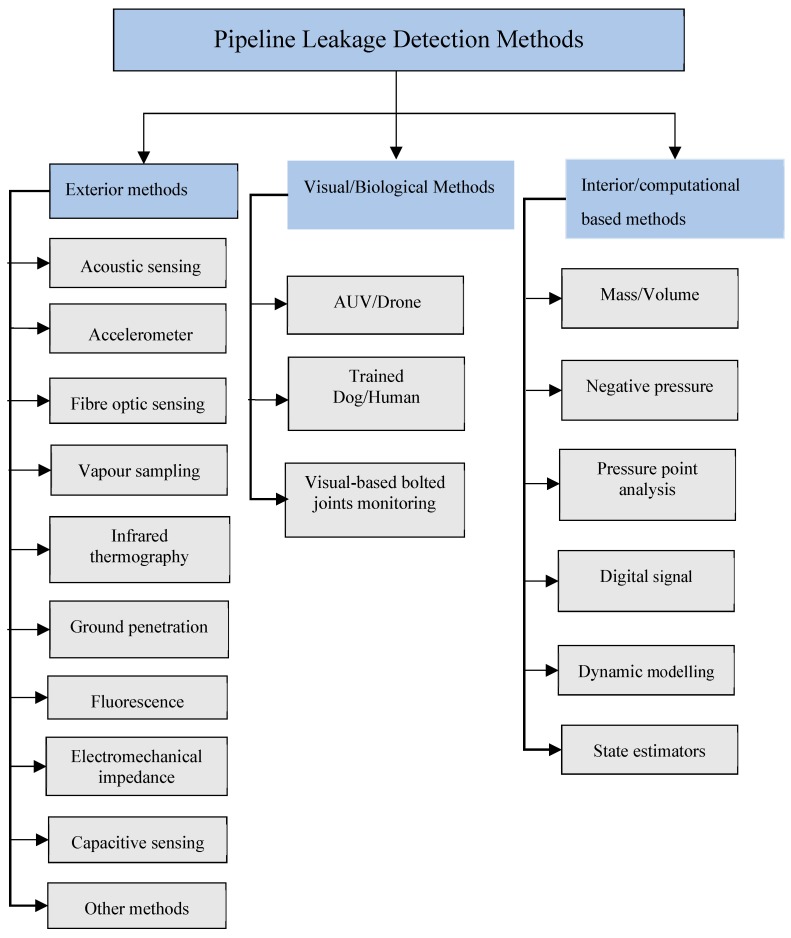
Flow chart of different pipeline leakage detection approaches.

Several pipeline leak detection methods have been proposed during the last decades using different working principles and approaches. Existing leakage detection methods are: acoustic emission [13,14,15], fibre optic sensor [16,17,18], ground penetration radar [19,20], negative pressure wave [21,22,23], pressure point analysis [24,25,26], dynamic modelling [27,28], vapour sampling, infrared thermography, digital signal processing and mass-volume balance [29,30,31,32,33]. These methods have been classified using various frameworks. Some authors have classified them into two categories: hardware and software-based methods [34,35]. In an attempt to group these methods based on technical nature further research efforts have been made [36,37,38,39,40,41,42] which has led to the classification of available leakage detection systems into three major groups, namely internal, non-technical or non-continuous and external methods [2,4]. In this study, we will classify different methods into the following categories: exterior, visual or biological, and interior or computational methods. A detailed classification of these methods is shown in Figure 2. The exterior approach utilises various man-made sensing systems to achieve the detection task outside pipelines. Moreover, the biological approach utilises visual, auditory and/or olfactory senses of trained dogs or experienced personnel to detect leakage. In addition, the interior approach consists of software based methods that make use of smart computational algorithms with the help of sensors monitoring the internal pipeline environment for detection task. Remote monitoring can be achieved by carrying camera or sensing systems to designated locations by smart pigging, helicopter or Autonomous Underwater Vehicles (AUVs)/drones or using sensor networks [2].

This paper aims to examine the state-of-the-art achievements in pipeline leakage detection technologies and to discuss research gaps and open issues that required attention in the field of pipelines leakage detection technology. The rest of the paper is organised as follows: Section 2 presents the exterior-based leak detection methods and compares their strengths and weaknesses; Section 3 presents the visual/biological-based leak detection methods; Section 4 presents the interior- based leak detection methods and features their strengths and weaknesses. The comparative performance analysis of the reviewed methods is given in Section 5. Section 6 gives the guideline for selecting an appropriate leak detection method for various operating environments. The research gaps and open issues on pipeline leakage detection and characterisation are discussed in Section 7. Finally, a summary of this paper, and possible future directions are presented in Section 8.

## 2. Exterior Based Leak Detection Methods

Exterior methods mainly involve the use of specific sensing devices to monitor the external part of the pipelines. These methods can be used to determine abnormalities in the pipeline surrounding and also detect the occurrence of leakages. Irrespective of the working principles these sensing methods are based on, they require some form of physical contact between the sensor probes and the infrastructure under monitoring. Examples of these devices include acoustic sensing, fibre optic sensing, vapour sampling, infrared thermography and ground penetration radar. The operational principle, strengths and weaknesses of these methods are discussed in the subsequent sections.

### 2.1. Acoustic Emission Sensors

Acoustic emission, according to American Society of Mechanical Engineering (ASME) 316 standard [43], is defined as “the class of phenomena whereby transient elastic waves are generated by the rapid release of energy from localised sources within a material, or the transient waves so generated”. Acoustic emission employs noise or vibration generated as a result of a sudden drop in pressure to detect the occurrence of pipeline leakage. When a pipeline leak occurs, it generates elastic waves in the frequency range up to 1 MHz [44] due to high-pressure fluid escaping from the perforated point that allows one to detect pipeline leakage incidents [4]. The time lag between the acoustic signals sensed by two sensors is employed to identify the leakage position [45]. Acoustic methods for leak detection can be divided into two classes [46]: active and passive. Active methods detect pipeline defects by listening to the reflected echoes of sound pulses emitted due to leakage. On the contrary, passive methods detect defects by listening to changes in sound generated by pressure waves in the pipelines. There are three major categories of acoustic sensors namely aquaphones, geophone and acoustic correlation techniques. Aquaphones require direct contact with hydrants and/or valves, while geophones listen to leaks on the surface directly above the pipeline. At the same time, steel rods can also be inserted into the buried pipe to transmit signals to mounted sensors on the rods. The amplitude of the measured pressure signal is measured as Sound Pressure Level (SPL) [2]:

(1) $$SPL=20\log_{10}\left(\frac{P}{P_{0}}\right)$$

where $P_{0}$ represents a reference sound pressure amplitude which is generally taken as 20 μPa [47]. As SPL is directly proportional to the gas generated power due to expansion it can also be expressed as:

(2) $$SPL\propto \log_{10}\left(\frac{RT}{M}\dot{m}\right)$$

where R is the gas constant, T is the gas temperature at the orifice, M is the molecular weight and $\dot{m}$ represent jetting gas mass flow rate. Both aquaphones and geophones can be used to detect and locate leakage. However, these approaches are not effective due to their slow operating procedures [48]. The acoustic correlation method is more sophisticated than the abovementioned methods. In this approach, two sensors are required to be positioned on either side of the pipe to detect leakage. The time lag between the acoustic signals when the sensors sense a leak is used to detect and identify the point of leakage [45,49]. 

The use of acoustic emission methods for pipeline leaks detection have been reported in several studies [50,51,52]. A reference standard for setting up and evaluating acoustic emission sensors deployed for pipeline leakage detection was proposed by [53]. The authors’ aimed to develop a reference standard for acoustic signals in order to provide a valuable threshold for checking out monitoring infrastructure and characterising source mechanisms to quantify leakages based on acoustic emission technology. By introducing several kind of controlled leakages, the effect of pressure and air injection were determined for thread leakages on the order of $0.1{\;\mathrm{galhr}}^{-1}$. Experimental investigation of pipeline leakage subjected to socket joint failure using acoustic emission and pattern recognition was proposed in [54]. The study showed that dominant frequency of environmental noise is less than 2 kHz while the dominant frequency of the acoustic signals due to the failure of the socket joint is concentrated in the 0–10 kHz range. The feature set obtained was trained with an artificial neural network (ANN) and good estimation accuracy of 97.2% and 96.9% was achieved. This indicates that acoustic emission-based methods can exhibit high sensitivity over long distances. However, additional strategies to increase the leak noise (such as pipeline pressure amplification) may be required. In [55], a combination of Linear Prediction Cepstrum Coefficient (LPCC) and Hidden Markov Model (HMM) was devised to examine the damaged acoustic signals. The HMM was used to identify corrupted signals while LPCC which represents short-time acoustic signals was adopted as the characteristic signal parameter. The obtained results revealed that the acoustic signal recognition rate was improved up to 97%.

Jia et al. conducted a gas leakage detection experiment on a gas pipeline length of 3.13 km using measured acoustic waves with the sensors positioned at different locations along the gas pipeline [17]. During the experiment, it was observed that acoustic waves generated due to the leakage transmitted from the rupture point to all sides of the pipeline at the rate of gas velocity, but the high frequency components of the acoustic decayed much faster than the low-frequency counterparts did. Therefore, they concluded that it is sufficient to detect leakage in gas pipelines using low-frequency signals. Applying acoustic emission for detecting leakage on pipeline networks can achieve early leaks detection, estimation of leak sizes and leak point localisation [39]. However, the effect of background noise can easily mask the actual sound of a leak. In order to overcome this challenge, several signal analysis techniques have been proposed in literature such as interrogation methods [13], wavelet transform methods as well as the combination of acoustic sensors with other types of sensors such as magnetic flux leakage [14,52,56].

The use of a cross-correlation method for detecting multiple leaks points in buried pipelines was investigated in [57]. The study revealed that measuring acoustic emission signals using two detectors positioned at both side of the pipe was efficient. Noise elimination and feature extraction on weak leak signatures using wavelet entropy was proposed in [58]. The weak signal was revealed using nonlinear adaptive filtering in accordance with the different characteristics between the actual signal and noise. Chen et al. demonstrated that small pipe leaks signal can be efficiently differentiated from noise and effectively localised. Oh et al. [51] have proposed an acoustic data condensation approach to determine and condense the distinguishable feature from the acoustic signals data so that high-pressure steam leakage can be diagnosed effectively. The obtained results showed that the proposed method successfully enable reduced sets of data to characterise the acoustic signature. Generally, the benefit of using acoustic emission for monitoring of pipeline network are easy utilisation of interrogation as well as the convenience of installation as it does not require system shutdown for installation or calibration. However, background noise can easily mask the sound of leakage at a high flow rate so that critical leakage may not be detected reliably.

### 2.2. Accelerometers

Apart from the abovementioned studies that are totally based on acoustic emission, accelerometers are another type of vibro-acoustic measuring device that are also useful to monitor low-frequency pipe-shell vibrations [59]. Several studies have been proposed for achieving leak detection and localisation using accelerometers [60,61]. El-Zahab et al. [60] utilised wireless accelerometers to detect leakage events on the exterior of valves connecting pipeline networks. Experimental investigation of cross-spectral density of surface vibration measurement at discrete locations along the pipelines such as junctions, bends and different pipeline sizes using accelerometers was proposed in [61]. The use of both accelerometers and hydrophones for monitoring pipelines was proposed in [62,63]. The obtained results showed that satisfactory leak detection performance was achieved on both transducers.

### 2.3. Fibre Optic Method

**Figure 3:**
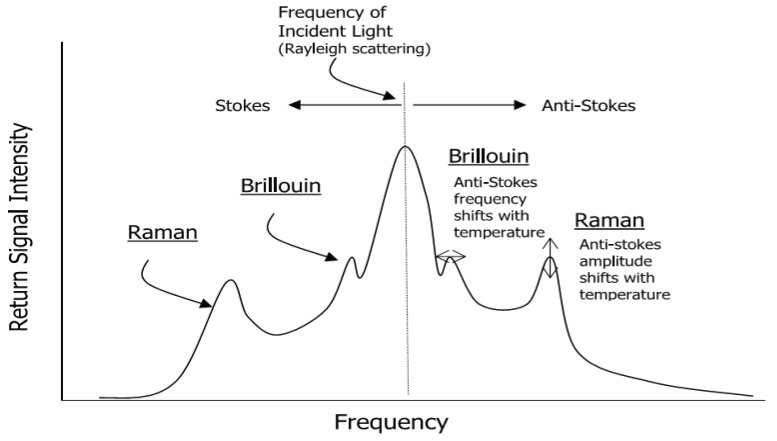
Schematic representation of the electromagnetic spectrum illustrating Rayleigh, Brillouin and Rayleigh scattering [66].

This method involves installation of fibre optic sensors along the exterior of the pipeline. The sensors can be installed as a distributed or point sensor to extensively detect the variety of physical and chemical properties of hydrocarbon spillage along the pipelines. The operation principle of this method is that cable temperature will change when pipeline leakage occurs and hydrocarbon fluid engross into the coating cable. By measuring the temperature variations in fibre optic cable anomalies along the pipeline can be detected [4]. Distributed Optical Fibre Sensor (DOFS) provides environmental measurements based on three classes of scattering, namely Raman, Rayleigh and Brillouin scattering [64]. These classifications are based on the frequency of the optical signals as illustrated in Figure 3. Brillouin scattering can measure both strain and temperature but is very sensitive to strain, while Raman scattering is only sensitive to temperature, with greater ability to accurately measure temperature greater or equivalent to 0.01 °C resolution [65].

The manifestation of Brillouin scattering takes place as a result of the interaction between acoustic waves and propagated optical signals. This leads to a shift in frequency components in the received light, but in the case of Raman scattering approach changes in temperature only result in backscattered light intensity fluctuations. The frequency shift mechanisms in Raman backscattered light consists of two components, namely, Stoke and anti-Stoke components [66]. The variation in temperature does not affect the amplitude of the Stoke components, while the amplitudes of anti-Stoke components vary dynamically in accordance with temperature changes. The operation method of Rayleigh scattering is based on elastic scattering (i.e. scattering without frequency variations) where the scattered power is directly proportional to the incident power which makes it attributable to non-propagation density fluctuations [67]. Brillouin scattering can be measured based on spontaneous or simulated ways; however, identification of the wavelength shift of the scattered light acts as a key means of measuring Brillouin scattering [66]. One of the benefits of pipeline leakage detection using fibre optics is the ability to detect small leaks [64]. Moreover, the potential of monitoring long pipelines and capability to accurately functioning in both subsea and surface pipeline networks can also be considered as another benefit of fibre optic-based systems [4]. However, its shortcomings include short lifespan and the inability to estimate the rate of leakages. Besides, the installation of fibre optics system over a large and complex pipeline network is challenging as optical fibres are fragile.

Several pipeline leakage detection systems based on fibre optic approaches have been proposed in literature [5,68,69,70]. The effectiveness of using distributed optical fibre for pipeline leak detection has been reported in [71]. In general, optical fibre is used for dual functions: signal transmission and sensing. The leak position is determined using the time order of the anti-Stoke light received at the measuring station. A similar study based on a macro-bend coated fibre optic was proposed by [72]. In this study, bending structure and macro-bending loss was utilised as a sensing mechanism for leak detection. The incidence of leaks was determined through comparison of the power loss at a different bending radius, and wrapping turns number. The obtained result revealed that the proposed system was able to detect leakages at the frequency range of 20 Hz to 2500 Hz. In [17] the authors have implemented the loop integrated Mach-Zehnder interferometer for an optical fibre-based vibrational sensor in pipeline monitoring and leakage localisation. The implemented system was tested with a 40 m steel field pipeline and the obtained results show good performance, with an average percentage error of 0.4% and 2.64% at 2 bar and 3 bar of pressure, respectively [18].

Water ingress which commonly occurs in a low-pressure gas pipeline distribution network is a major challenge in subsea pipeline systems [73]. This occurs whenever groundwater enters the pipeline through a crack point and blocks the flow channel. In an effort to detect and determine the location of water ingression, a temperature distribution sensing mechanism based on fibre optics was experimentally studied in [65]. The observed alterations in temperature from the distributed sensors were utilised for detecting the presence of water ingress. Subsequently, the variations in cable temperature were employed to determine the window of interest which indicates the location of the leakage. The outcome of this study indicates that distributed optical fibre sensors are capable of detecting water ingress accurately, even if the water ingression position is dynamically changing. A recent study reported the design of a distributed Fibre Bragg Grating (FBG) hoop strain measurement system in combination with a support vector machine algorithm for continuous gas pipeline monitoring as well as leakage localisation [5]. In this study, various kernel function parameters are optimised through five-fold cross-validation to ensure the highest leak localisation accuracy.

### 2.4. Vapour Sampling Method

Vapour sampling is generally used to determine the degree of hydrocarbon vapour in the pipeline environment. Though, it is applicable in gas storage tank systems, it is also suitable to determine gas discharges into the environment surrounding the pipeline. The tube is pressure dependent and filled with air at atmospheric pressure. Oil spillage can be determined by measuring the recorded gas concentration as a function of the pumping time for thus the degree of absorption [38]. In the events of pipelines leakages, vapour or gas diffuses into the tube as a result of concentration gradient which, after a certain period, will generate an accumulated signal indicating hydrocarbon flit in the tube environment [37]. As the gas concentration increases the leak peak also increases. The higher the gas concentration in the tube surrounding, the more the leak peak increases. 

**Figure 4:**
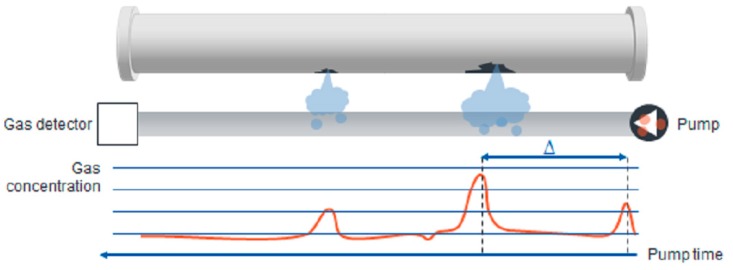
Sensor hose system for pipeline leakage detection [2].

Different types of vapour sampling-based pipeline leak monitoring systems have been proposed in the literature [35,74]. The use of sniffer tubes based on a hydrocarbon permeable cylinder for detecting spillage around the pipeline environment was reported in [75]. According to the study in [2], a sensor hose must be positioned underneath then pipeline to detect gas diffused out of pipes due to leakage. Figure 4 illustrates sensor hose positioning in the pipeline for maximisation of the system effectiveness.

The advantages of vapour sampling systems include the capability of detecting small leaks, independent of the pressure or flow balance and superlative performance for detecting leaks in multiphase flow applications [39]. Besides, the sensor can withstand significant hydrostatic pressure. However, one of the major deficiencies of this technique is the response time. Usually, it takes several hours to days to respond to leaks [74]. Therefore, coupling a vapour sensor with another leak detection method will provide better response time.

### 2.5. Infrared Thermography

**Figure 5:**
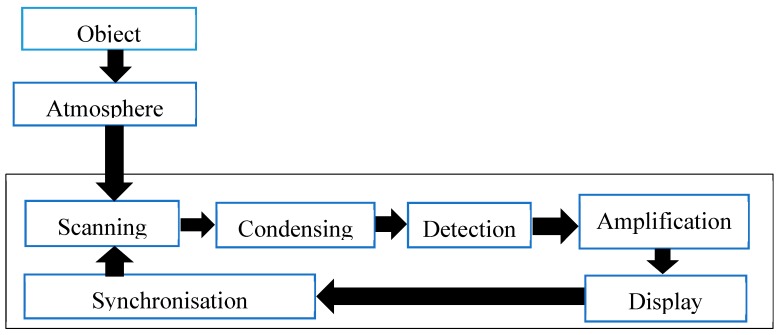
Basic functions of a IR thermography camera [30].

Pipeline leakage detection systems based on the infrared thermography (IRT) mechanism are also applicable for the detection of pipelines leakages. IRT is an infrared image-based technique that can detect temperature changes in the pipeline environment using infrared cameras which shows the infrared range of 900–1400 nm [30]. The image captured using an IR thermography camera is referred to as a thermogram. The basic function of thermography cameras is illustrated in Figure 5. Since changes in temperature measurements are one of the common indications of gas discharge in the pipelines surrounding as gas leaks usually cause abnormal temperature distribution, therefore, using IRT for pipeline monitoring become widely accepted due to its capability to measure temperature changes in real-time and in a non-contact manner [76,77]. IRT as a contactless and non-invasive condition monitoring tool is also applicable for various condition monitoring applications such as heat transfer [78], tensile failure [79], concrete and masonry bridges [80].

**Figure 6:**
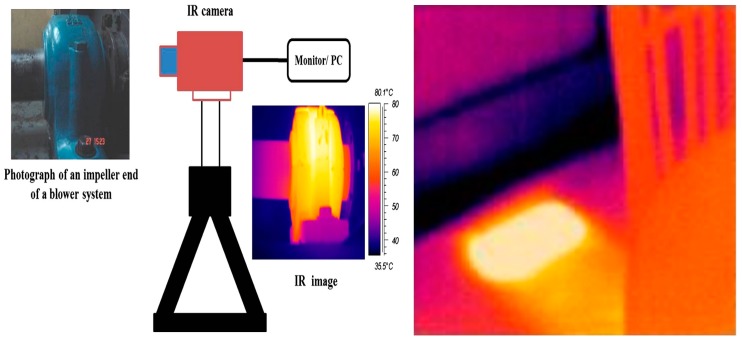
Experimental setup of IRT based system for anomalies monitoring [30,76].

Thermal cameras are effective devices for sensing objects of various shapes with different material properties from any perspective. The object acquired using a thermal camera can be processed to recognise anomalies in the pipeline environment through the warm and cooler areas displayed in the thermal image with a different colour in that particular environment. Figure 6 illustrates the experimental setup of a typical IRT-based system for anomaly detection in a pipeline environment. Thermography can be divided into two categories [30]: active and passive thermography. Active thermography features the area of interest with the background thermal contrast, while the area of interest is focused on temperature variation and background in passive thermography. Unlike other temperature measurement mechanisms such as resistance temperature detectors (RTDs), and thermocouples, IRT provides contactless, non-invasive, real-time and distributed measurement of temperature across a continuous region. IRT can remotely measure the temperature distribution of an object and provide a visual image that indicates the degree of the data measured in that region with different colours.

Improvements of IRT have been ongoing for several decades. Details of the origin and theory of IRT have been presented in [81]. IRT has been widely used in pipeline monitoring [82,83,84]. An innovative method of detecting pressure air and gas leakages using passive IR thermography was reported in [69]. A similar work reported in [30] proposed a pipeline leaks detection system using the IR thermography technique. In their approach, the fundamental principle of IRT was used to differentiate various kinds of anomalies from thermal images using basic image segmentation algorithms to distinguish the defect areas in the images. This work concluded that cavitation erosion, piping clogging and steel tanks can be inspected using an infrared camera. In the study of [84], a method for gas leak detection based on a thermal imaging approach was proposed. The pipeline surroundings were inspected using an infrared camera followed by filtering processing where the targeted region of interest was enhanced and segmented to extract features suitable for identification of rupture areas in the pipelines. The designed system demonstrated the ability to distinguish between normal and abnormal gas pipeline conditions.

The use of IRT systems for pipeline condition monitoring enables timely detection of anomalies in the pipeline network thereby, reduced loss associated with gas wastage. Besides, the complexity of IRT system integration is not high. The major components to set up the system are a camera stand, an infrared camera and a display unit for visualisation of the acquired infrared thermal images. Moreover, the benefits of the IRT system include efficient transmission of the scan objects into a visualisation form [85], fast response time, and ease of use [86]. The operation of such systems is so straightforward that no specially trained or experienced personnel are required for the monitoring task. IRT-based systems are suitable for any kind of pipeline size as well as various hydrocarbon fluids flowing through the pipelines [87]. However, the cost of a high resolution infrared camera is very expensive. Moreover, quantifying a leak orifice of less than 1.0 mm using IRT-based systems is challenging.

In an attempt to address these shortcomings, a leakage quantification mechanism using a combination of infrared thermography and ultrasound methods was proposed [87]. The reported results indicate that thermography is appropriate to quantify pipeline orifices larger than 1.0 mm, while ultrasound was proved to be usable for all orifice dimensions. A similar study reported in [88] combined thermal images (thermograms) and a platinum resistance temperature detector (RTD) method to achieve accurate spot temperature measurements. The study employed an experimental flow rig with an internal diameter of 50 mm and the volumetric rate of the leakage was determined using numerical computation. The leak flow through the crack in mass was quantified using the following equation [88]:

(3) $$Q=\alpha A\varphi_{max}\sqrt{2P\rho }$$

where A is the cross-sectional area of the crack in m^2^, α represents an area correction factor, ρ represents the gas density function in kg/m^3^, P is the absolute pressure in Pa and $\varphi_{max}$ is the maximum leak rate and is computed to be 0.4692. The flow dispersion function $\varphi_{max}$ for $CO_{2}$ gas from the pressurised enclosure was computed as:

(4) $$\varphi_{max}={\left(\frac{2}{\gamma +1}\right)}^{1/\left(\gamma -1\right)}\sqrt{\frac{\gamma }{\gamma +1}}$$

where γ represents the specific heat ratio of $CO_{2}$. The $CO_{2}$ gas density is adopted from the ideal gas law and is presented as:

(5) $$\rho =\frac{P}{Rco_{2}T}$$

where $R_{CO_{2}}$ represent specific gas constant of $CO_{2}\left(=188.9\;J/kgK\right)$ and T represents flow temperature in degrees Kelvin. 

### 2.6. Ground Penetration Radar

The emergence of ground penetration radar (GPR) is considered as an environmental tool which is valuable to detect and identify physical structures such as buried pipelines, water concentrations and landfill debris in the ground [89]. The use of GPR technology for underground monitoring is particularly useful to aid mine detection efforts which can be traced back to 1960 [90]. GPR is a non-invasive high resolution instrument which utilises electromagnetic wave propagation and scattering techniques to detect alterations in the magnetic and electrical properties of soil in the pipeline surrounding [91]. Detection of subsurface objects using then radar approach was first proposed by Cook in 1960 [92]. Readers are referred to [93] for the basic working principles of GPR. In order to detect subsurface object reflection levels, Moffatt and Puskar [94] reported an improved radar-based object detection mechanism for the investigation of man-made objects. An electromagnetic wave speed in any medium is dependent upon the speed of light (c) in free space (c = 0.3 m/ns). The speed of electromagnetic wave ($V_{m}$) in a given material can be determined as follow [95]:

(6) $$V_{m}=\frac{c}{\sqrt{\left(\varepsilon_{r}\;\mu_{r}/2\right)\left(\left(1+P^{2}\right)+1\right)}}$$

where $\varepsilon_{r}$ is the relative dielectric constant of the material, $\mu_{r}$ is the relative magnetic permeability of the material ($\mu_{r}$ = 1 for non-magnetic material. P is the loss factor, such that $P=\frac{\sigma }{\omega \varepsilon }$, σ is the conductivity, ω=2πf (where f is the frequency in Hz) and $\varepsilon =\varepsilon_{r}\varepsilon_{o}$ (where $\varepsilon_{o}$ is the free space permittivity ($8.85\times 10^{-12}$ F/m). In low-loss materials, loss factor P≈0, and speed of electromagnetic wave is given as:

(7) $$V_{m}=\frac{c}{\sqrt{\varepsilon_{r}}}=\frac{0.3}{\sqrt{\varepsilon_{r}}}\;\left(m/ns\right)$$

By first determining the medium velocity ($V_{m}$) using Equations (6) and (7). The penetration depth (D) of electromagnetic wave can be computed as follows:

(8) $$D=\frac{\sqrt{{\left(T.V_{m}\right)}^{2}-S^{2}}}{2}$$

where S represents the fixed distance between the transmitting and receiving antennas of the GPR system, and T is the travelling time history of the GPR signal. The contrast in the relative dielectric constant between adjacent layers is a function of electromagnetic radiation. The proportion of the reflected energy given as reflection coefficient (R) is determined as:

(9) $$R=\frac{V_{1}-V_{2}}{V_{1}+V_{2}}$$

where $V_{1}$ and $V_{2}$ represent the velocities in layer 1 and layer 2, respectively, of the medium, and $V_{1}$ is smaller than $V_{2}$. Additionally, the reflection coefficient can also be determined as:

(10) $$R=\frac{\sqrt{\varepsilon_{r2}}-\sqrt{\varepsilon_{r1}}}{\sqrt{\varepsilon_{r2}}+\sqrt{\varepsilon_{r1}}}$$

where $\varepsilon_{r1}$ and $\varepsilon_{r2}$ represent the relative dielectric constants of layer 1 and layer 2, respectively.

The GPR has proved impressive potential as an effective non-destructive tool for detecting underground objects [96]. However, GPR signals can be easily corrupted by environmental noise [19]. In order to overcome this shortcoming and enhance GPR profile features, several signal processing approaches have been reported in [97,98]. Zoubir et al. [99] proposed a Kalman filter for detection of landmines using impulse ground penetration radar. An improvement of this study using a particle filter was proposed in [100]. In order to remove false alarms in GPR systems, a novel cluster suppression landmine detection algorithm based on a correlation method was reported in [101]. 

Bradford et al. implemented oil spill detection in and under snow assessment using an airborne GPR technique [102]. In this study the authors observed that oil located underneath snow tends to reduce the impedance contrast with core ice and results in anomalous low amplitude radar reflections. The outcome of this research revealed that, by using 1 GHz GPR system, a 2 cm dense oil film trapped between sea ice and snow can be detected with a 51% reduction in reflection force. The authors reported that this approach shows better performance even though in the presence of weak signal to noise ratio (SNR). Besides, GPR-based pipeline leak detection systems are highly suitable for underground pipelines, as they are reliable and provide detailed information of subsurface objects. However, they are not applicable for long pipeline networks. The effectiveness of IRT may be significantly reduced for buried pipelines, depending on the depth of the pipe and the the use of covering media such as concrete. Similarly, the operation is limited in a clay soil environment as iron pipe corrosion materials can hide cast iron pipelines from the GPR. Hence, for the GPR to be effectively operated an adequate bandwidth is required for the detected signal at the desired resolution and noise levels. Effective coupling of electromagnetic radiation in the ground, and sufficient penetration of the radiation through the ground regardless of targeted depth is of paramount importance. 

### 2.7. Fluorescence Method

Fluorescence methods for hydrocarbon spill detection employ light sources of a specific wavelength for molecule excitation in the targeted substance to a higher energy level [46]. The detection of the spill is based upon the proportionality between the amount of hydrocarbon fluid discharged and rate of light emitted at a different wavelength which can then be picked up for detection of occurrence of hydrocarbon spillage. Detection of leakages has been successfully implemented using fluorescent dyes (unfiltered ultraviolet) light [103]. Since fluorescence detectors have high spatial coverage capability, quick and easy scanning can be performed by mounting the sensors on a ROV manipulator and the detection of leakages can be easily achieved regardless of tidal flow direction. However, if the concentration of the fluorescent dyes is very high, the visibility of the monitoring environment must be high to achieve optimal system performance. Another shortcoming of the fluorescent dyes, especially in underwater environments, is the effects of untuned black light that can easily mislead observers from tracking the leak location [104]. Although, this issue has been partially solved by developed submersible (tuned) fluorimeters that can transmit data up to attendant vessel to provide a real time display, this challenge still remains as an issue in turbid waters. 

### 2.8. Capacitive Sensing 

In this technique the change in the dielectric constant of the medium surrounding the sensor is measured to identify existence of hydrocarbon spillage [46]. The capacitive sensor is a local coverage point sensor which is generally employed in subsea pipelines. The sensors use the variations in dielectric constants between seawater and hydrocarbons to detect existence of hydrocarbons which cause an imbalance in measured capacitance once it gets in contact with the sensor. Sensor sensitivity with respect to the leak size is dependent of the distance between the leak position and the drift of the leaking medium [105]. Capacitive sensor has been introduced to the market for environmental monitoring [106]. However, a numbers of false alarms have been reported from the operator [105]. The causes of these errors may largely be due to the fact the sensor requires direct contact with the leaking medium. Besides, buoyancy effects may carry the leaking medium away from the sensor vicinity which can be overcome by installing a collector for hydrocarbon spills over the monitoring structure.

### 2.9. Electromechanical Impedance-Based Methods 

In electromechanical impedance (EMI)-based techniques a variation in structural mechanical impedance instigated by the incidence of pipeline damage is monitored to detect the occurrence of pipeline failure. EMI transducers are made up of small piezoelectric patches that is usually less than $25\times 25\times 0.1\;{\mathrm{mm}}^{3}$ and their dynamic impedance is measured for leak detection [107]. In the event of pipeline defects, the EMI employs high-frequency structure excitation (usually greater than 30 kHz) through a surface-bounded piezoelectric sensor to sense variations in structural point impedance. Over the years, the EMI method has been a great interest to monitoring different structures, including pipelines. The feasibility of utilising impedance-based assessments for pipeline structures was proposed in [108]. Zuo et al. [109] proposed a modified EMI approach for detecting the incidence of cracks that comprises fusing signals from multiple transducers. In the study of Xu et al. [109] a new method of pipeline defect localisation using time-reversal and a matching pursuit algorithm was proposed. One of the major advantages of using EMI for monitoring pipeline structures is its capability to utilise a single piezoelectric transducer to act as both sensor and actuator. However, due at lower Curie temperatures, it is difficult for the EMI method to be conducted in an environment with high temperature [110]. In order to overcome this shortcoming, a new method to avoid attaching the piezoelectric transducer directly onto the targeted structure using frequency shifting to compensate for signature changes was proposed by Na and Lee [110]. The obtained result showed that identification of structural defects can be accurately achieved in an environment above 200 °C temperature.

### 2.10. Other Methods

This section briefly presents less popular methods for pipeline leaks detection techniques based on information provided by the Joint Industry Project (JIP) offshore leak detection industry [46], equipment suppliers [111], using some of these technologies and some of the available literature [112]. Techniques covered include spectral scanners, Lidar systems and electromagnetic reflection. Spectral scanners are passive sensors that analyse solar light reflected by a material. It detects pipeline leakages by comparing spectral signatures against a normal background. Lidar systems use pulsed laser radiation as the illumination source to determine the presence of methane. The absorption of the energy by the laser along the pipeline length is determined using a pulsed laser detector. The emitted energy at different wavelengths is measured through electromagnetic reflection. Electromagnetic reflection and other leak detection mechanisms such as ultraviolet scanner, microwave radiometer and visual surveillance cameras are regarded as passive monitoring devices that work through detecting either the radiation emitted by leaked natural gas or the background radiation. This makes passive-based systems less expensive in general. Table 1 provides a summary, strengths and weaknesses of the exterior leak detection techniques.

## 3. Visual/ Biological Leak Detection Methods

Visual/biological methods of detecting leakages refer to the traditional process of detecting oil spillage in pipeline surroundings using trained dogs, experienced personnel, smart pigging or helicopters/drones [2]. This method usually utilises trained personnel who walk along the pipelines and search for anomalous conditions in the pipelines environment. Trained observers can recognise the leaks through visual observation or smelling the odour coming out from crack point. Similarly, the noise or vibrations generated as oil escapes from rupture point also applicable in this method to detect and locate pipeline failures. Both dogs and smart pigging function in a similar way to the experienced personnel. The pig is sometimes equipped with sensors and data recording devices such as fluorescent, optical camera or video sensors with great sensing range if the visibility level is high. A trained dog is more sensitive to the odour of certain gases than human beings or pigging in some cases [113,114]. Conversely, dogs are not effective for prolonged operation for more than 30–120 min of continuous searching due to fatigue [115]. These on-site inspection methods can only be applied to onshore or shallow offshore pipeline networks. Besides, the detection time is also based on the frequency of inspections which normally takes place in some countries such as the USA for at least once every three weeks [35]. The recent development of remotely operated vehicles (ROVs) has transformed the operation style of offshore oil transportation operators. It has been shown that ROVs are durable for performing subsea pipeline inspection tasks and functioning in deep water that cannot be accessible by dog, pigging or human divers [116]. The operation principle of ROVs is based on teleoperation that involves a master-slave system. The slave is a ROV which is designed to interact with the extremely hazardous subsea environment while the master human operator is located in a safe place to remotely control the slave robot’s motions using input devices, like joysticks or haptic devices [117]. All robot commands, sensory feedback and power are sent through an umbilical cable connecting the ROV and the deployment vessel.

**Figure 7:**
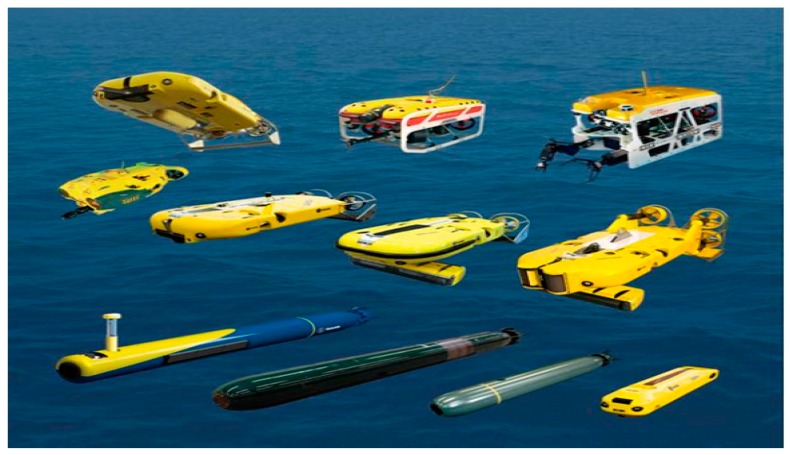
Different kinds of AUVs and ROVs [116].

The emergence of autonomous underwater vehicles (AUVs) in subsea pipeline inspection and monitoring has reduced the extent of human operator involvement in unmanned vehicles through the implementation of intelligent control machinery and thus drastically lower the chance of human casualties. Though, the operation principle of AUVs is similar to the teleoperation of ROVs, only limited skilled operators are required in supervisory control of AUVs [118]. There are numerous types of AUVs and ROVs available for oil and gas infrastructural monitoring. Examples of commercially available ROVs and AUVs primarily deployed in the oil and gas industry are shown in Figure 7. The use of unmanned vehicles for pipeline inspection has the advantage of being a remote operating system; making it suitable for inspection in a remote and hazardous environment. Lower cost of maintenance and higher operation safety are also some of the advantages of unmanned vehicles. Unfortunately, these systems also have drawbacks. For example, the cost of purchase or hiring an AUV/ROV is extremely high. Additionally, bad weather conditions such as clouds, winds or other climatological agents can restrict the performance of these vehicles. There are also legal constraints for the use of the unmanned system in some certain areas due to safety concerns because unmanned vehicles usually lack onboard capacity to sense and avoid other AUVs in advance [119]. However, great effort has been spent on underwater robot sensing and navigation research to realise fully autonomous AUVs for pipeline inspection and monitoring tasks with minimal human intervention. [120,121].

As bolt connections are widely utilised in the assembly of different sections of petroleum pipeline systems, effective technique for monitoring bolted flange connections is essential. Several vision-based assessment methods for real-time bolt looseness detection have been proposed [122,123,124]. Nguyen et al. [125] proposed a vision-based algorithm to identify bolt-looseness in steel structure bolted flange connections. A similar vision-based monitoring technique for detection of bolted joints looseness in wind turbine tower structures was proposed by Park et al. [126] which can be adopted to pipeline monitoring in a fairly straightforward fashion. Wang et al. [127] proposed a new vision-based bolts looseness detection method to address the issues of difficulty in detecting the status of bolt image acquired from any arbitrary perspective and high performance bolt looseness recognition model. The algorithm developed shows high capability to identify the mark on the bolt and bolt position on the flange connection in offline mode. In order to enhance the robustness of this method further online training is required. Similarly, the method should be improved to be able to recognise bolts looseness in a pool of large flag bolts.

## 4. Interior/Computational Methods

Interior or computational methods utilise internal fluid measurement instruments to monitor parameters associated with fluid flow in pipelines. These systems are used to continuously monitor the status of petroleum products inside the pipeline such as pressure, flow rate, temperature, density, volume and other parameters which quantitatively characterise the released products. By fusing the information conveyed from internal pipeline states, the discrepancy between two different sections of the pipeline can be used to determine the occurrence of leakage based on various methods, namely mass-volume balance, negative pressure waves, pressure point analysis, digital signal processing and dynamic modelling. Details of each of these techniques are discussed in the subsequent sections.

### 4.1. Mass-Volume Balance

The mass-volume balance approach for leak detection is straightforward [128]. Its operation is based on the principle of mass conservation [129]. The principle states that a fluid that enters the pipe section remains inside the pipe until it exits from the pipeline section [130]. In a normal cylindrical pipeline network, the inflow and outflow fluid can be metered. In the absence of leakage, the assumption is that the inflow and outflow measured at the two ends of the pipeline section must be balanced, so a discrepancy between the measured mass-volume flows at the two ends of the pipeline indicates the presence of a leakage. The inconsistency of the values in measurement can be determined using the principle of mass conservation given as follows [37]:

(11) $$\dot{M_{i}}\left(t\right)-\dot{M_{o}}\left(t\right)=\frac{dM_{L}}{dt}$$

where $\dot{M_{i}}\left(t\right)$ and $\dot{M_{o}}\left(t\right)$ represent the mass flow rate at the inlet (i) and outlet (o), respectively. The mass stored across the pipeline length is denoted by $M_{L}$, while L represents the length of the pipeline section. In a cylindrical pipeline system, the mass stored $M_{L}$ for a pipeline of length L changes over time as a result of changes in fluid density (ρ) and cross-sectional area (A) satisfies Equation (12):

(12) $$\frac{dM_{L}}{dt}=\frac{d}{dt}\overset{L}{\underset{0}{\int }}\rho \left(x\right)A\left(x\right)dx=\overset{L}{\underset{0}{\int }}\frac{d}{dt}\langle \rho \left(x\right)A\left(x\right)\rangle dx$$

where ρ(x)A(x)dx represent the differential mass stored across the length of the pipeline $\left(M_{L}\right)$ and ρ changes in accordance to the relation; ρ(x)A(x)dx is measured with coordinate position x, 0≤x≤L. If ρ and A is assumed to be constant, $\frac{dM_{L}}{dt}=0$. Then Equation (12) becomes:

(13) $$\dot{M_{i}}\left(t\right)-\dot{M_{o}}\left(t\right)=0$$

Similarly, according to [106], assuming $\rho_{i}\left(t\right)=\rho_{i}=\rho_{l}$ and $\rho_{o}\left(t\right)=\rho_{o}=\rho_{l}$ are equal and constant for inlet and outlet mass flow, by introducing volume flow $\dot{V}$ with $\dot{M}=\rho \dot{V}$ then:

(14) $$\dot{V_{i}}\left(t\right)-\dot{V_{o}}\left(t\right)=0$$

The imbalance (R) between inlet and outlet volume can be estimated and compared as given in (15) and (16) respectively:

(15) $$\dot{R}\left(t\right)\dot{=}\dot{V_{i}}\left(t\right)-\dot{V_{o}}\left(t\right)$$

(16) $$R=\{\begin{array}{cc} <R_{th} & in\;absence\;of\;leak\\ \geq R_{th} & if\;there\;is\;a\;leak \end{array}$$

where $R_{th}$ is a threshold to evaluate the imbalance of the volume between inlet and outlet volume.

This method has been commercialised and is widely adopted in the oil and gas industry [38]. Some of the existing flow meters in the industry include orifice plate, positive displacement, turbine and mass flow devices. Some scientific papers based on this method have been reported in the literature [131,132]. A robust means of detecting leakage in pipeline networks using the mass imbalance technique was proposed in [133]. In this study, the activities of calibration and prediction were unified to infer the presence and characterise leakages. A similar study [132] reports a mass balance compensation method for oil pipeline leak detection systems comparing the difference in mass at the two ends against mass balance experiments. The obtained result showed that the proposed system can function in various pipeline networks under different operating conditions. The occurrence of leakage with a low rate of change in pressure or flow rate can be detected using this method. However, one of the biggest limitations of this method is the uncertainty inherent to the instrument. It is sensitive to random disturbances and the dynamics of the pipelines [29]. Besides, the inability to locate the position of leakage is another disadvantage of this method. Nevertheless, a hybrid of mass balance and other leaks detection techniques will enhance the effectiveness of the system. In addition, by increasing the number of measuring devices along the pipeline, localisation of points of leakage will be achieved.

### 4.2. Negative Pressure Wave

Leak detection techniques using negative pressure waves (NPWs) are based on the principle that when a leakage occurs, it causes a pressure alteration as well as a decrease in flow speed which results in an instantaneous pressure drop and speed variation along the pipeline. As the instantaneous pressure drop occurs, it generates a negative pressure wave at the leak position and propagates the wave with a certain speed towards the upstream and downstream ends of the pipe. The wave contains leakage information which can be estimated through visual inspection and signal analysis to determine the leakage location by virtue of the time difference with which the waves reach the pipeline ends [134]. A NPW-based leakage detection technique is cost-effective as it requires little in the way of hardware in the whole pipeline network to detect and locate leaks. 

This method has been widely employed in pipeline monitoring due to its fast response time and leak localisation ability [135]. However, it is only effective for massive instantaneous leaks and easily leads to false alarms due to the difficulty in differentiating between normal pressure waves and leakages. Similarly, precise determination of the leak location using time difference in pressure wave detection at the two ends of pipeline is another critical challenge of this method. In order to alleviate this shortcoming, several efforts have been devoted to improving the leak detection and localisation mechanisms using NPW [23,136,137]. Identification of the signal that indicates a leak and normal pipeline operation using structure pattern recognition was proposed by [138]. The use of adaptive filters and Kalman filters for extraction of pressure wave inflexion information was proposed in [139] and [140]. In the study of [141], a negative pressure wave signal analysis system based on a Haar wavelet transformation was proposed. The authors demonstrated an effective way of detecting signal variations in the pressure wave signal and established a systematic way of using wavelet de-noising schemes to overcome the destructive noise attenuation problem.

The pressure wave signal created by small leakage can be easily mixed with noise and background interference. This makes accurate signal detection and thus the oil spillage detection process challenging. An effective method of identifying small leakage signals using an improved harmonic wavelet was proposed in [142]. The proposed scheme was used to extract the pressure wave signal from the background noise, but the shortcoming of this approach is the decay rate of pressure wave signal in time domain. In order to address this issue, the authors adopted a window function to smooth the harmonic wavelet. Different methods of addressing the effect of background interference from leakage signals have been proposed in the literature. An independent component analysis (ICA) technique for separation of the characteristic signatures of the pressure wave signal mixed with the background noise was reported in [143]. A similar study proposed an improved robust independent component analysis method for effectively separating mixed oil pipeline leak signals [144]. The proposed method was based on statistics estimation and iterative estimation technique using information theory.

**Figure 8:**
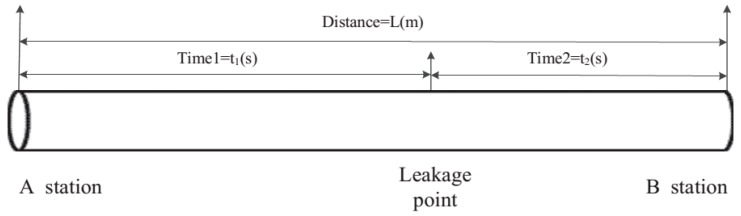
Negative pressure wave monitoring system [147].

An alternative method of detecting small leakages using a specially designed morphological filter has been presented in [21]. The morphological filter was employed to filter background noise and retain the basic geometry features of the pressure signals. A time reversal pipeline leakage localisation approach using an adjustable resolution mechanism was proposed in [145]. The proposed scheme formulated a method of fine-tuning leak localisation resolution in an interval of time. An experimental study on leakage localisation based on dynamic pressure waves was proposed in [146]. In that study, an improved wavelet transform approach was developed, and the theoretical propagation model of dynamic pressure waves was established. Similarly, Li et al. proposed detecting negative pressure waves with an intelligent machine learning technique using a moving windows least square support vector machine [147]. The parameter of interest in the study centred on wave arrival time from the leak point to the end sides of the pipeline (i.e. $t_{1}\left(s\right)$ and $t_{2}\left(s\right)$) using negative pressure wave signals and sensor positioning principles as shown in Figure 8.

The location of a unknown leakage along the pipeline between stations (sensors) A and B shown in Figure 8 is determined using mathematical models (17) and (18) [147]:

(17) $$t_{1}-t_{0}=\overset{X}{\underset{0}{\int }}\frac{1}{a_{X}-V}dX$$

(18) $$t_{2}-t_{0}=\overset{L}{\underset{X}{\int }}\frac{1}{a_{X}+V}dX$$

where X(m) is the distance from leak position to the sensor A, L(m) represents the distance from sensor A to B, $a_{X}\;\left(m/s\right)$ represents the propagation velocity of the negative pressure wave in the pipeline, $t_{0}$ is the time leak occur and V (m/s) is the liquid velocity. Assuming that the time difference in which the wave travelled from the first station to the end of the sensor is represented as $\Delta t=t_{1}-t_{2}$, the above equations were reformulated and given as:

(19) $$\Delta t=\;\frac{X}{a-V}-\frac{L-X}{a+V}$$

where a is the velocity of negative pressure wave and X is the distance from a leak point to the pressure sensor A. When the fluid temperature, density and elasticity of the negative pressure wave propagation change, the fluid velocity will also change accordingly, due to this, the negative pressure wave velocity was formulated and given as:

(20) $$a=\sqrt{\frac{k/\rho }{1+\left(k/E\right)\left(D/e\right)C}}$$

where $\rho \;\left(\mathrm{kg}/m^{3}\right)$ is the liquid density, k (Pa) is the liquid bulk modulus of elasticity, E (Pa) is the modulus of elasticity, C is the correction factor related to the pipeline constraints, and e (m) is the pipeline thickness.

### 4.3. Pressure Point Analysis

The pressure point analysis (PPA) method is a leak detection technique based upon the statistical properties of measured pressures at different points along the pipeline. The leakage is determined through the comparison of the measured values against the running statistical trend of the previous measurements [148]. If the statistical pressure of the new incoming data is considerably smaller than the previous value or smaller than a predefined threshold, it indicates a leakage event. This method is considered as one of the fastest ways of detecting the presence of leakage in a pipeline based on the fact that existence of leak always results in an immediate pressure drop at the leakage point [8,35]. 

The PPA method has been successfully applied in underwater environments, cold climates and functions adequately under diverse flow conditions. Small leakages which cannot be easily detected by other methods can be detected using PPA. However, it is difficult to determine leak location using this method [65]. The ease of use and low cost of implementation are the major advantages of this method, but in a batch process where valves are opened and closed simultaneously, transient states may arise and create a period which may easily lead to a false alarm. In order to overcome this drawback, the operation changes must be defined so that detection of leakage can be restrained pending the steady state operation returns to the pipeline. Similarly, integrating this method with other techniques such as mass-volume balance improve its effectiveness. 

### 4.4. Digital Signal Processing 

**Figure 9:**
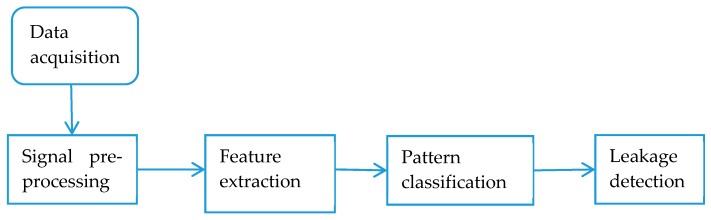
The architecture of pipeline leaks detection based on digital signal processing.

In digital signal processing approaches, the extracted information such as amplitudes, wavelet transform coefficients and others frequency response is employed to determine leakage events. Generally, pipeline leak detection using digital signal processing involves five steps as illustrated in Figure 9. The steps are as follows: (1) initially internal sensors measure in-pipe pressure or flow; (2) After data acquisition, the acquired data is pre-processed to filter the background noise for efficient feature extraction; (3) In the feature extraction step, various statistical, spectral and signal transform techniques are employed to extract relevant features to monitor the state of hydrocarbon fluid transport in the pipeline; (4) The pattern of the extracted feature is compared with the known pre-set signal or previous features for decision making; (5) Leakage detection is achieved through the comparison of the pattern with the threshold.

Different signal processing techniques have been employed in this research domain. Some of the existing method includes wavelet transform [149,150], impedance method [151], cross-correlation [32] and Haar wavelet transform [141]. Shibata et al. devised a leakage detection system using Fast Fourier transform (FFT) [152]. The proposed method detects pipe leak positions through analysis of the data obtained at a certain distance from the leakage point. The classification and discrimination of the orifice signals are carried out based on the obtained signal patterns. Lay-Ekuakille et al. proposed a spectral analysis of the leak detection system in a zig-zag pipeline using the filter diagonalisation method (FDM) [153]. That study aimed to utilise the FDM as an improved alternative to the Fast Fourier transform (FFT) to minimise the FFT recovery error in a narrow pipeline network. Santos-Ruiz et al. proposed an online pipeline leakage diagnosis system based on an extended Kalman filter (EKF) and steady state mixed approach [154]. The efficiency of the method was evaluated using online detection, localisation and quantification of non-concurrent pipeline leakages at different positions. The obtained results indicated an average error estimate of less than 1% of the flow rate and 3% of the leak localisation.

The authors of [155] proposed a small leak feature extraction and recognition scheme for natural gas pipelines using local mean decomposition envelope spectrum entropy to decompose the leak signal into product function components. Based on the obtained kurtosis features, the principal product function components with higher leak information content were chosen for further processing. Sun et al. proposed a hybrid ensemble local mean decomposition (ELMD) and sparse representation for recognition of leakage orifices in a natural gas pipeline [31]. In that study, an ELMD scheme was employed to perform adaptive decomposition of the leak signatures and acquisition of information feature of the leak signal based on different orifice scenarios. An application of phased antenna arrays for improving resolution in detecting leakage was proposed in [12]. Xiao et al. [3] proposed a hybridisation of cross-time-frequency spectrum and variational decomposition analysis for natural gas pipeline leakage localisation. The variational mode decomposition was used to decompose leakage signals into mode components, while the adaptive selection model based on mutual information was utilised to process the mode components to obtain feature closely related to the leak signatures. The proposed system was experimentally validated and the results obtained revealed that average relative localisation errors can be reduced dramatically.

A recent study by Liu et al. proposed a new method of leak localisation for the gas pipeline using acoustic signals [156]. Two methods based on the amplitude attenuation model using a combination of wavelet transform and blind source separation was proposed to address the challenges of leak localisation. The authors observed that when the decomposition level of the signal increases, the contribution to localisation error by leak time deviation and amplitude decreases. It also revealed that the combined methods are effective in solving the signal attenuation problems for pipeline leakages. The advantage of digital signal processing techniques is their simplicity in implementation as pipeline leakages are detected through sophisticated algorithms for leakage data signature (in time, frequency, or both domains) extraction running on common embedded computing hardware or digital signal processors (DSPs). However, the main challenge associated with this approach is the detection accuracy as the acquired data is usually attenuated and contaminated by noise. Besides, in order to effectively detect leakages, a large sensor network is required to cover the whole pipeline network.

### 4.5. Dynamic Modelling 

Dynamic modelling-based pipeline leak systems are gaining considerable attention as they appear to be a promising technique for the detection of anomalies in both surface and subsea pipeline networks. In this approach, mathematical models are formulated to represent the operation of a pipeline system based on physics principles. The detection of leakages using this method is performed from two different points of views: (1) a statistical point of view and (2) a transient point of view. From the statistical point of view, the system utilises decision theory based on the assumption that parameters associated with fluid flowing remain constant except in the presence of anomalies along the pipeline [157]. Hypothesis testing involved for detecting leakage is based on the uncompensated mass balance through the utilisation of either single or multiple measurements carried out at different time instants.

According to the technical report of the Alaska Department of Environmental Conservation [158], the most sensitive, but also most complex pipeline leak detection technique in use is the transient leakage detection technique. Detection of leakage in pipelines mainly requires the formulation of a mathematical model using fluid flow equations. The equations of state for modelling fluid flow includes the equations of conservation of mass, conservation of momentum, conservations of energy and states of the fluid. This method requires measurements of flow, temperature, pressure and other parameters associated with fluid transport at the inlet and outlet of the pipeline or at several points along the pipeline. The transient event or noise levels are continuously being monitored using discrepancy between the measured values and simulated values to detect the occurrence of leakages. Transient-based leak detection approaches have been proposed by the research community in various studies [26,27,159,160,161]. 

Yang et al. proposed a characterisation of hydraulic transient modelling using the equations of state of the fluid [27]. Partial differential equations that modelled the dynamics of fluid flow in the pipeline are simplified into ordinary differential equations using a fixed grid to represent the numerical solution at some discrete points. A similar method of detecting pipeline leakage using flow model analysis was proposed in [162]. In that study, a mathematical model was formulated to predict the flow distribution of soil gas through porous probes at various positions on the horizontal sampling line. A computational fluid dynamics (CFD)-based method was proposed to describe the underwater gas release and dispersion from subsea gas pipeline leakages [28]. The simulation was based on an Eulerian-Lagrangian modelling concept to predict the released gas plume by considering bubbles as discrete particles. The simulation result was validated against experimental data, and the obtained results revealed that CFD model could provide valuable output in subsea pipeline leakage detection such as gas release rates, horizontal dispersion distances and gas rise times. However, it can only be applicable in shallow ocean areas as the sea waves can easily alter the gas dispersion movements. Besides, in the event of large leakages, the gas release rate and dispersion pattern vary. Therefore, the deviation of parameters associated with gas product transport in subsea pipelines appears to provide useful information that can reveal the state of hydrocarbon fluids in subsea gas pipeline networks. Similarly, according to the report of Pipelines and Installations-RU-NO Barents Project [163], a suitable subsea pipeline leak detection system should be able to provide information about the internal flow condition without being affected by the subsurface ice situation as well as ocean activity such as seawaves, ocean currents and so on. 

### 4.6. State Estimators/Observers Method

State estimator or observer is a method that is based on dynamical modelling of flow process in the pipeline to estimate or observe variations in the variables associated with the fluid flow and indicate the occurrence of fault as a result of pipe damage [164]. This technique in a usual sense can be regarded as an auxiliary dynamical model for estimation of the internal parameters of a flow process [164]. The state observers have been employed to reconstruct the state vector and estimate the missing variables in the flow process [165]. A general review of recent observers in applied chemical process system was undertaken by Ali et al. [165] who presented six different kinds of state estimators, including: Luenberger-based observers, finite-dimensional system observers, Bayesian estimators, artificial intelligence based observers, disturbances and fault detection observers and hybrid observers. The details and evaluation of these observers based on their attributes, merits and limitations can be found in [165,166]. Further classifications and applications of each of observers such as extended Luenberger observer, sliding mode observer, extended Kalman filter, etc. were also presented [165,166]. Modelling of pipeline failures using some of these classes has been proposed in the literature [167,168,169]. 

A leak detection and isolation algorithm that is based on state estimation observer was proposed in [167]. The observer was designed as an extended Kalman filter on the basis of a discretised model both in time and space. Besançon et al. [168] proposed a direct observer model for detecting and estimating leaks in pipelines. In their approach, a simple way of obtaining more efficient model was formulated using finite-dimensional approximation. Modisette [169] observed that using a Kalman filter to estimate flow process in a large dynamic system such as typical pipeline model is computationally intractable. In an attempt to improve pipeline modelling with greater computational effectiveness, a new approximate form of the Kalman filter that based on the Ensemble Kalman filter (EnKF) was designed. The study implemented an EnKF for state estimation on artificial gas and liquid pipelines with known errors, and compared it to existing techniques of state estimation and automatic tuning for speed and accuracy. The obtained results revealed that the proposed model produced good estimations. More studies on applying the Kalman filter, extended Kalman filter and Ensemble Kalman filter in pipeline leak detection can be found in [170,171,172,173,174,175].

Torres et al. [176] investigated the use of both a high gain observer and an extended Kalman filter for monitoring pipeline flow processes. Experiments were carried out in two phases: the first phase considered the leak detection and isolation problem and the second phase investigated friction estimation in the process. A similar study in [177] proposed a high gain observers method to detect and isolate leakages in subterranean liquefied petroleum gas (LPG) pipelines. The authors connected a sub-observer in cascaded form in order to generate residuals that allow the detection of leakages and isolating the region where a leakage occurred in the pipeline. Apart from the above study, more studies on pipeline monitoring systems using sliding model observers were also presented in [178,179,180,181]. Hybridisation of temperature variation parameter modeling and extended Kalman filtering for reliable leak diagnosis was proposed by Delgado-Aguinaga et al. [182]. In this study, the effects of temperature changes on fluid properties such as friction factor, water density and bulk modulus were considered. In a related study of Begovich et al. [183], the effects of fluid temperature on performance of a classical flow modeling on plastic pipe were also investigated, but their study was only limited to influence of temperature variations in the water viscosity and friction factor. Table 2 provides a summary of the strengths and weaknesses of the interior leak detection approaches.

## 5. Performance Comparison of Leak Detection Technologies

**Figure 10:**
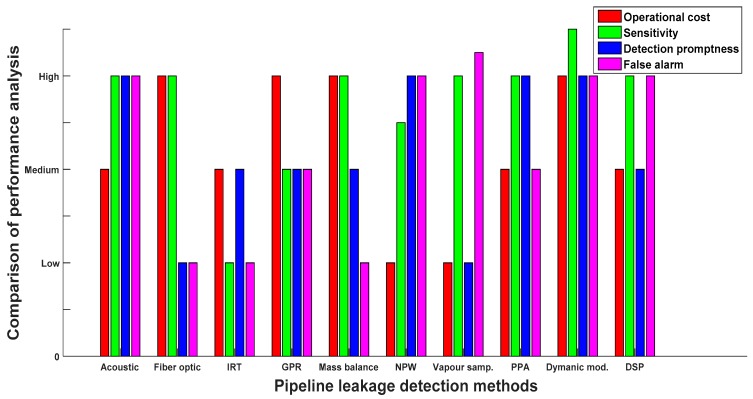
Three level performance analysis comparison.

This section presents a qualitative performance analysis of various pipeline leak detection approaches based on the literature cited above and American Petroleum Institute (API) performance requirement guidelines [4,91]. Various performance criteria are considered for comparison such as system operational cost, sensitivity, accuracy, leak localisation, system mode of operation, ease of usage, leak size estimation, ease of retrofitting and false alarm rate. The analysis is performed using two and three-level performance comparison. In the three-level analysis comparison, the operational cost, sensitivity and false alarm rate are compared in the range of low, medium and high. Figure 10 shows abar chart representing the three-level analysis of the reviewed methods based on their unique strengths and weaknesses. As shown in Figure 10, most of the techniques have high operational cost except NPW and vapour sampling. However, the high rate of false alarms is the major weakness of these two methods. In general, all methods perform well in terms of sensitivity, except IRT, GPR and NPW. The rate of false alarms in most of the techniques such as acoustic emission, NPW, vapour sampling, dynamic modelling and DSP are high. Though many researchers have been working on alleviating these drawbacks, reducing false alarms in acoustic emission and DSP appears to be a challenging task as acoustic emissions are highly sensitive to random ambient noise and the DSP approach mainly depends on instrument calibration accuracy. Besides, different circumstances such as pipeline corrosion, bending and blockage can easily lead to false alarms in DSP. Among all the reviewed methods, the dynamic modelling method shows high sensitivity in detecting the presence of pipeline leakages. However, the high complexity of the mathematical models involved and strict experienced personnel requirements are the key challenges of this method. With the help of recent advances in high performance computing and cloud computing technologies, the dynamic modelling approach will become more and more popular in the oil and gas industry.

The performances of various pipeline leakage detection methods are next compared using two-level performance analysis. System accuracy, system mode of operation, leak localisation, leak size estimation, ease of usage and ease of retrofitting are the criteria employed to evaluate the performance of the reviewed methods using a yes or no, high or low, and steady or transient state or not applicable (indicated by “—”) scale. Table 3 shows a summary of the comparison. The study shows that none of the methods satisfies all attributes as they all vary in merits and critical shortcomings. For example, the systems based on infrared thermography are proved to be better in terms of system accuracy, leak localisation, easy usage and easy retrofitting, however, estimation of the leakage rate is difficult with this method. Similarly, almost all methods satisfy the ease of retrofitting or upgrading criterion except the fibre optic sensing method, where a point of breakage can lead to total system failure thereby requiring total sensor network replacement. System accuracy is also an important criterion to evaluate the performance of a pipeline leak detection system. Although some of the methods perform better in regards to this criterion, system detection capability also depends on other factors such as instrument calibration, and the quality and quantity of the instruments used.

## 6. Guideline for Pipeline Leakage Detection Method Selection

As mentioned in the previous sections, there are various methods and mechanisms for pipeline leak detection and localisation. However, the applicability of each method varies considerably depending on pipeline operating conditions, pipeline characteristics and the medium to be detected. For instance, detection of leakages in surface, underground or subsea environments can be attained through the use of the approaches such as fiber optic cable, fluorescence and interior methods. While GPR can only be applied to underground pipeline networks. Some methods are applicable for all types of hydrocarbon fluid—including oil and gas—and water. However, only specific types of hydrocarbon fluid can be detected by some methods. In order to provide guidelines for selecting a method appropriate for a particular scenario, Table 4 lists the major available leak detection techniques and guidelines for their selection. The information is based on review works in the literature and information provided by the Joint Industry and Project (JIP) offshore leak detection report [44]. “Local coverage” refers to the small area within the vicinity of the sensor. While “Area coverage” means that sensor can cover a large area but not entire field coverage.

## 7. Research Gaps and Open Issues

Based on the various reviewed pipeline leak detection methods, research gaps and future research directions are identified in this section. The performance of pipeline leakage detection methods generally varies depending on the approaches, operational conditions and pipeline networks. However, guidelines set by American Petroleum Institute (API 1555) such as sensitivity, accuracy, reliability and adaptability [92] must be met before we can consider any leak detection system suitable for production solutions. Moreover, leak localisation and estimation of the leakage rate are also important as they will facilitate spillage containment and maintenance at an early stage to avoid serious damage to the environment. The simplest way to achieve this goal is through deployment of a vast number of leak detection sensors in a sensor network between the upstream and downstream of the pipeline. By doing so, it will easy to isolate the leak position and thus improve the ability to track when a sensor acquires anomalous information at the expense of high implementation cost. 

Remote monitoring of oil and gas pipeline networks using wireless communications technology provide benefits of low cost, fast response and the ability to track the locations where leakages occur. However, to attain benchmark performance in monitoring pipelines remotely some of the design issues that require research attention include sensing modality, sensing coverage and leak localisation. As mentioned in the previous sections, several sensors are designed for monitoring pipeline leakages using different sensing modalities. Usually, sensors are deployed for monitoring steady-state conditions where the physical pipeline context is expected to remain stable over time. Variations in physical parameters of the pipeline operation such as vibration, temperature, pressure etc. are expected to be detectable and communicated to reveal the incidences of anomalies. Leaks can only be accurately detected if the incident is within the vicinity of the monitoring sensor and thus the accuracy of leak detection systems becomes questionable if the leaks are not within the receptive fields of the sensors. Sensors deployed for remote monitoring of pipelines are employed to perform both sensing and communication functions, however, the challenge of how to cover a monitoring region efficiently and relay the obtained measurements to their neighbouring nodes is also challenging in wireless sensor networks (WSNs), which impact on the network performance can be severe. There are many issues in designing optimal WSNs, particularly for pipeline monitoring. These issues include: (i) self-organisation, (ii) fault-tolerance, (iii) optimal sensor node placement, (iv) sensor coverage, (v) energy saving routing, (vi) energy harvesting and so on. 

During the lifetime of the sensor network some of the deployed sensor nodes are expected to experience hardware failure and the network may not be able to cope with this failure. This will limit the effectiveness of the whole network. The operation and performance of WSNs is largely dependent on optimal node placement as communication among the sensor node is required to transmit the acquired data. Besides, sensor placement also influences the resources management such as energy consumption in WSNs [184], while the energy consumption influences the network lifetime [185]. In that case, sensor placement in pipeline monitoring requires further research attention. The development of self-organisation strategies has become an important research issue in WSNs. Sensor nodes are smart enough to autonomously reorganise themselves to share sensing and data transmission tasks when some nodes fail. The issue of coverage problems has been addressed in the literature [186,187,188]. Some of these studies have proposed methods for achieving high sensor coverage [189,190,191], while development of analytical model and optimisation approaches for WSN coverage was proposed in some studies [192,193,194]. However, the development of simple but realistic models for analysis and optimization still remains as a challenging research questions. Since a high percentage of pipeline systems are made up of underground and underwater pipelines networks and the power required for real-time sensing and data communications in such environments is demanding, better replacement of sensor nodes in these settings is expensive or infeasible for large sensor networks. In order to achieve long-lived networks in these energy constrained environments, different energy consumption minimisation methods such as low energy adaptive clustering hierarchy [195], in-network processing [196], and sleep mode configuration [197] have been applied. Energy can also be harvested from the resources in the pipeline surroundings such as fluid flow, pipe vibration, pressure and water kinetics using piezoelectric transducers. Although great improvements have been observed in research and development of wireless sensor network technology, efficient and reliable energy storage and generic plug and play energy harvesters from multiple sources remain open research challenges.

Leak localisation is very essential in pipeline monitoring as it will speed up the repair process. Different methods of defect localisation in pipelines have been proposed [198,199,200]. The performance of these techniques, however, varies in terms of accuracy, degree of complexity and operation environments. Mobile sensor nodes with built-in Global Positioning System (GPS) have been successfully deployed to determine and report the geographical location of pipeline leakages. The use of mobile sensor nodes in pipeline environments is essential as it can enhance coverage and recover the network from any failure which partitions the whole network into multiple disconnected subnetworks. However, the cost of implementation of these sensor nodes with GPS capability is extremely high. Besides, it may be difficult for the GPS signals to penetrate the metal or concrete walls which protect pipelines. If all sensor nodes are static, their locations are marked using GPS and stored permanently in a map in the deployment phase. Leaks can then be localised based on the known locations of reporting sensor nodes. On the contrary, scalability of the pipeline leakage detection sensor network is another research challenge when the coverage of the pipeline network is huge. In this regard, localization techniques with satisfactory performance will be a welcome addition to the leak detection mechanism toolbox. The effect of temperature variation which is a common type of environmental uncertainty, affects the accuracy of flow monitoring systems significantly. Environmental uncertainties can affect the properties of fluids in pipelines such as fluid density, viscosity, friction factor, etc. Although, some studies have provided insights for the development of temperature-dependent flow models [182,183], these investigations are only limited to short flow models in which spatial changes of the temperature can be neglected. A robust temperature variation compensation approach will provide additional advantages for fluid flow modeling. It is important to detect the valid leaks and reduce the number of false positive alarms so that pipeline leak detectors can attain acceptable accuracy. All leakage detectors are based on inference based on evidence acquired from sensors [201]. The input evidence signature is usually noisy or error prone. The noise is in general random in nature and its underlying probability distribution is unknown. The source of the noise comes from inaccurate system measurements, instruments calibration, system modelling, data processing, feature extraction as well as communications. For example, in an acoustic emission leak detection method data acquired using acoustic sensors noise disruption as well as signal attenuation phenomena are usually inherent. In order to reduce the effect of this noise, certain design requirements for signal filtering must be met. Effectiveness of some of the signal filtering algorithms such as Savitzky-Golay, Ensemble, Applet [202] can lessen the degree of signal distortion to acceptable level. An autonomous system which can detect, locate and quantify the rate of leakage with the capability to manage a large amount of acquired data is essential for planned and unplanned leak incidents. Advanced data visualisation tools will definitely help in showing the state of flow activities for decision making in leak detection, localisation and characterisation, and pipeline maintenance. In addition, data driven self-testing incidents analysis and other offline performance validation methods will also enhance the system flexibility. 

The subsea industry activity has been continuously growing, which has made the sector a truly global industry with the industry operations amounting to billions of NOK in turnover [203]. However, pipeline leakages remain one of the major challenges in this sector [204] although various efforts have been made to guarantee early detection of leakages in subsea pipelines. In [163], computational fluid dynamics modelling was devised to describe underwater gas release and dispersion trajectories. The challenges of this approach are that seawaves can easily alter the gas dispersion movements and in the event of large leakages, the gas release rate and dispersion trajectory could be arbitrary. The mechanistic modelling of detection pipeline leaks at a fixed inlet rate presented in [24] provides insights for monitoring hydrocarbon parameters. However, the algorithm is limited because the external conditions that can easily lead to subsea pipeline instability in a subsea environment were not taken into consideration. Updated information about the internal flow conditions as well as pipeline integrity that are independent of the weather and sea conditions is needed for further innovation in this area. Moreover, experiment leak scenarios as a function of leak opening size in the laboratory and data processing in a way suitable to establish signals indicating hydrocarbon spillage will provide benefits in designing a functional basis for leak detection. 

In general, the aim of future pipeline monitoring is to design a real-time intelligent pipeline leak detection and localisation system for subsea pipeline networks. The effect of environmental factors, in particular, hydrodynamic forces due to oblique wave and current loading on subsea pipelines still require further research study. Extensive simulation and laboratory experiments are being conducted to study the effects of leakage parameters, like size and shape, on the flow mechanism and validate different models. Numerical simulations of fluid flow in pipeline using computational fluid dynamics (CFD) have been proved to provide a better understanding of pipeline internal flow and the conditions of pipeline leaks on various scales, thereby reducing the cost in experimental studies. However, high computational complexity remains one of the major drawbacks of CFD. Further research efforts are still required to optimise and/or parallelise CFD solution algorithms in terms of computation and memory resource constraints.

## 8. Summary and Conclusions 

This survey paper provides a rudimentary reference to guide readers in selecting an appropriate leak detection technology for a particular setting. In this paper, a comprehensive survey of various available pipeline leakage detection and localisation methods was carried out. A summary of what has been demonstrated to date is presented, along with research gaps and open issues that require attention in this research domain. A wide variety of pipeline leak detection approaches was reviewed and grouped into three different categories. The first category is the exterior methods which involve the use of specially designed sensing systems to monitor the external parts of pipelines. The methods considered in this category includes acoustic emission sensors, fibre optic sensors, vapour sampling, infrared thermography and ground penetration radar. In the second category, the visual methods of detecting leakages in the pipeline which include trained dogs, experienced personnel, smart pigging or helicopters/drones/ROVs/AUVs were discussed. The interior method of detecting leakage using parameters associated with hydrocarbon fluid such as mass-volume balance, negative pressure waves, pressure point analysis, digital signal processing and dynamic modelling were presented in the third category. We then performed a comparative analysis using various performance requirements based on the American Petroleum Institute (API) guidelines [4,92]. Based on the analysis, it can be concluded that each technique has some merits and drawbacks. For example, most of the interior methods are sensitive to small leakages, especially if the point of leakage is close to the sensing device, but they are more prone to false alarms as they can easily be affected by environmental noise. Mass-volume balance and numerical computation models exhibit good performance for high flow rates, in multiphase flow and subsea pipeline networks. Finally, we discussed the research gaps and open issues in pipeline leakage detection, characterisation and localisation. We observe that despite having invested a considerable amount of research effort in pipeline leak detection and localisation systems, various gaps must still be filled before a reliable real-time leakage detection in pipelines can be fully achieved. 

## Figures and Tables

**Table 1:** Summary of exterior pipeline leak detection methods.

Methods	Principle of Operation	Strengths	Weaknesses
Acoustic Emission	Detect leaks by picking up intrinsic signals escaping from a perforated pipeline.	Easy to install and suitable for early detection, portable and cost-effective.	Sensitive to random and environmental noise, prone to false alarms and not suitable for small leaks.
Fibre Optics Sensing	Detect leaks through the identification of temperature changes in the optical property of the cable induced by the presence of leakage.	Insensitive to electromagnetic noise and the optical fibre can act both as sensor and data transmission medium.	The cost of implementation is high, not durable and not applicable for pipelines protected by cathodic protection systems.
Vapour Sampling	Utilise hydrocarbon vapour diffused into the sensor tube to detect trace concentrations of specific hydrocarbon compounds.	Suitable for detecting small concentrations of diffused gas.	Time taken to detect a leak is long, not really effective for subsea pipelines.
Infrared Thermography	Detect leaks using infrared image techniques for detecting temperature variations in the pipeline environment.	Highly efficient power for transforming detected objects into visual images, easy to use and fast response time.	Quantifying leak orifices smaller than 1.0 mm using IRT-based systems is difficult.
Ground Penetration Radar	Utilise electromagnetic waves transmitted into the monitoring object by means of moving an antenna along a surface.	Timely detection of leakage in underground pipelines, reliable and leak information is comprehensive.	GPR signals can easily be distorted in a clay soil environment, costly and require highly skilled operator.
Fluorescence	Proportionality between the amount of fluid discharged and rate of light emitted at a different wavelength.	High spatial coverage, quick and easy scanning for leaks.	Medium to be detected must be naturally fluorescent.
Electromechanical Impedance	Utilise mechanical impedance changes deduced by the incident of pipeline defect.	A single piezoelectric transducer can serve as both sensor and actuator.	It is only applicable for metal pipelines, operational limitations in high temperature environments.
Capacitive Sensing	Measuring changes in the dielectric constant of the medium surrounding the sensor.	It can be employed for detection in non-metallic targets.	Requires direct contact with the leaking medium.
Spectral Scanners	Comparing spectral signature against normal background.	Capable of identification of oil type (light/crude) and thickness of the oil slick.	The amount of data generated by a spectral scanner is large which limited its ability to operate in nearly real-time.
Lidar Systems	Employed pulsed laser as the illumination source for methane detection.	Able to detect leaks in the absence of temperature variation between the gas and the surroundings.	High cost of execution and false alarm rate.
Electromagnetic Reflection	Measure emitted energy at different wavelengths.	It can indicate leak location	It can be affected by severe weather.

**Table 2:** Summary of the interior pipeline leak detection methods.

Methods	Principle of Operation	Strength	Weakness
Mass-volume Balance	Utilises discrepancy between upstream and downstream fluid mass-volume for determining the leakage.	Low cost, portable, straightforward and insensitive to noise interference.	Leak size dependent, not applicable for leak localisation.
Negative Pressure Wave	Utilises negative pressure waves propagated due to pressure drops as a result of leakage.	Fast response time and suitable for leak localisation.	Only effective for large instantaneous leaks.
Pressure Point Analysis	Monitor pressure variation at different points within the pipeline system.	Appropriate for underwater environments, cold climates and adequately functioning under diverse flow conditions.	Leak detection is challenging in batch processes where valves are opened and closed simultaneously.
Digital Signal Processing	Utilises extracted signal features such as amplitude, frequency wavelet transform coefficients, etc. from acquired data.	Good performance, suitable for detecting and locating leak positions.	Easily prone to false alarms, and can be masked by noise.
Dynamic Modelling	Detects leaks using the discrepancy between measured data and simulated values based on conservation equations and the equation of state for the fluid.	Applicable for leak detection and localisation, fast and a large amount of data can be handled.	High computational complexity, expensive and labour intensive.
State Estimation	Estimates the missing variables using a set of algebraic equations that relates a set of input, output and state variables.	Suitable for reconstruction of the state vector and estimating the missing variable.	The limitations vary based on estimator classes such as poor convergence factors, computational complexity, discarding of uncertainties during simulation etc.

**Table 3:** Two-level performance analysis comparison.

Methods	Performance Comparison Metric					
	System Accuracy	Leak Localisation	Leak Size Estimation	Ease of Usage	Ease of Retrofitting	Operational Mode
**Acoustic Emission**	High, but sensitive to random noise	Yes	No	Yes	Yes	-
**Fibre Optic Sensing**	High	Yes	Yes	Yes	No	-
**Vapour Sampling**	Depends on sensing tube closeness to spilled gas	No	No	Yes	Yes	-
**Infrared Thermography**	High	Yes	No	Yes	Yes	-
**Ground Penetration Radar**	Low	Yes	No	Yes	Yes	-
**Fluorescence**	Low	No	No	No	Yes	-
**Capacitive Sensing**	Low	No	N	Yes	Yes	-
**Mass-volume Balance**	Low, depends on instrument calibration and leak size	No	Yes	Yes	Yes	Steady state
**Negative Pressure Wave**	Low	Yes	No	Yes	Yes	Steady state
**Pressure Point Analysis**	Low	Yes	Yes	Yes	Yes	Steady state
**Digital Signal Processing**	Depends on leakage size and sensor used	Yes	No	Yes	Yes	Stead state
**Dynamic Modelling**	High, depends on pipeline stability and mathematical model	Yes	Yes	No	Yes	Both steady and transient state

**Table 4:** Summary of the guidelines for method selection.

Methods	Operating Environment	Sensor Coverage	Hydrocarbon Fluids
Acoustic sensing	All	Local	All
Fibre optic sensing	All	Local	All
Vapour sampling	Subsea	Local	All
Infrared thermography	All	Local	Oil and gas
Ground penetration radar	Underground	Local	Water and gas
Fluorescence	All	Local	Oil
Capacitive sensing	Subsea	Local	All
Spectral scanner	Surface	Local	Oil
Lidar system	Subsea	Local	All
Electromagnetic reflection	Surface	Local	Oil
Biological methods	Subsea	Local	All
Interior methods	All	Area	All
